# Tackling the perils of unawareness: the cluster headache case

**DOI:** 10.1186/s10194-017-0757-7

**Published:** 2017-04-27

**Authors:** Giorgio Lambru, Anna P. Andreou, Elena Ruiz de la Torre, Paolo Martelletti

**Affiliations:** 1 Headache Centre, Guy’s and St Thomas’ NHS Trust, London, UK; 2 Headache Research - Wolfson CARD, King’s College London, London, UK; 3 European Headache Alliance, Brussels, Belgium; 4 Regional Referral Headache Centre, Sant’Andrea Hospital, Rome, Italy; 5 Department of Clinical and Molecular Medicine, Sapienza University of Rome, Rome, Italy; 6 European Headache Federation, London, UK

## Cluster headache unawareness

The 21^st^ of March 2017 has marked a remarkable collective effort to raise public awareness of cluster headache (CH). This campaign has inspired an explosion of initiatives aimed to raise awareness of this devastating, yet neglected condition to the level of other neurological disorders. The collaboration between several stakeholders, namely the European Headache Federation [1], patient support groups such as the Organisation for the Understanding of Cluster Headache (OUCH) in the United Kingdom (UK) [2], the European Headache Alliance (EHA), the European Federation of Neurological Associations [3], the European Brain Council [4], many headache centres led by the Guy’s and St Thomas’ Headache Centre (London, UK) and the La Sapienza University Headache Centre at St Andrea Hospital, (Rome, Italy), Members of Parliament of the European Union and the UK, publishers [5] and the media [6], has provided a unique platform to discuss strategies to tackle the unmet need in CH. These bodies are all in agreement that the emphasis should be on: increasing awareness of CH; advancing the understanding and management of the condition; and ensuring standardised, high-quality care across Europe and the UK.

## Cluster headache is still lurking in the shadows

Although the clinical phenotype of cluster headache is typical and unmistakable, in practice, misdiagnosis is unfortunately common. Cluster headache is a quasi-rare primary headache disorder characterised by unilateral, sharp, stabbing pain rapidly reaching an excruciating intensity. The pain is typically felt around the orbit and temporal area and usually lasts between 15 and 180 min. The attacks are frequently described as feeling like a “red hot poker in the eye”. The pain is accompanied by at least one of the cranial autonomic symptoms [7]. Up to 93% of patients describe a sense of restlessness and agitation during the attacks [8]. The frequency of attacks ranges from once every other day up to eight episodes per day and, in episodic CH, are grouped in bouts lasting from a few weeks to a few months. They follow a striking tendency to circadian periodicity with a preponderance of attacks during the sleep phase. Furthermore, they may demonstrate a circannual periodicity, with active bouts peaking during seasonal changes, particularly the solstices and equinoxes (episodic CH). Unfortunately, approximately 10–20% of patients may develop the chronic form of CH, without any remission period of more than a month (chronic CH). Some people evolve from the episodic to the chronic form or vice-versa and some enter prolonged remission periods. However, the factors contributing to these pattern transitions are yet to be explained.

Despite its peculiar phenotype, only a small proportion of these patients are correctly diagnosed and many still face an average delay of 5.3 years before the correct diagnosis is made. Consequently, many sufferers may never receive the correct diagnosis and, of those who do, a significant minority still do not receive appropriate treatments [9]. Part of the problem is the lack of training in the medical schools, at the level of general practitioners and even higher, in the neurology specialty. There is little research assessing the burden of CH so it is difficult to express the impact that these inadequacies has on those with the condition, and the wider society. Large epidemiological projects are required to further clarify the disability associated with both the episodic and chronic forms of the condition and thus help CH gain the deserved recognition amongst other neurological disorders with a similar prevalence, such as multiple sclerosis [10]. The Global Burden of Diseases studies, which are acknowledged internationally as comprehensive reports in health metrics, morbidity and mortality, have yet to present any data on the burden and disability of CH [11].

The observance of the CH awareness day during the spring equinox highlights one of the many stereotypical features of this condition, which was known for over 350 years within the neuroscience community. The seasonal periodicity of CH has given the opportunity to disentangle the complex biological mechanisms of this fascinating brain disorder by focusing on neuronal structures that can produce stereotypical episodes of extreme head pain following a striking circadian and circannual periodicity. Indeed functional, structural and neuro-hormonal studies have confirmed the importance of the region that modulates fundamental circadian rhythms in mammals, namely the suprachiasmatic nucleus of the hypothalamus [12]. However, the factors that regulate the periodic derangements of the chronobiological activities relevant in CH is still far from understood. Resting state functional magnetic resonance studies have tried to shed light on networks relevant in CH by exploring functional connectivity changes in and outside CH bouts [13, 14]. Furthermore, several studies have tried to find a genetic signature that may increase the risk of developing CH, looking at pain processing, neuro-inflammatory and vascular markers, trigger factors, ion channels and clock genes, with little success [15, 16]. Recently, polymorphisms of the hypocretin receptor-2 gene (HCRTR2) have been associated with CH [17]. Together with the reduced cerebrospinal fluid levels of hypocretin-1 in CH patients [18], these data pointed towards the importance of an imbalance of the hypocretin system activity in CH and its possible role in modulating trigemino-vascular processes [19].

Lack of research funding has only allowed sparse studies aiming to unravel the pathophysiology of CH or the mechanisms of action of drugs that abort or prevent CH attacks. Part of the effort in raising awareness for this devastating disease includes the demand for specific funding streams in CH research. Further understanding of the pathophysiology of CH may stimulate the development of novel treatments targeting specific pathways for this disease. Indeed, for far too long the arsenal of treatments for CH has been limited to triptans and oxygen amongst abortive treatments, and verapamil, corticosteroids and lithium for CH prevention.

## Brightening the “cluster headache days”

Based on the promising data coming from studies using monoclonal antibodies against Calcitonin Gene-Related Peptide (CGRP) for migraine prevention, there are now randomised controlled phase III trials using these antibodies specifically for episodic and chronic cluster headache [20, 21]. Targeting CGRP in CH may offer new, more specific and potentially better-tolerated treatments. Advances in the neuromodulation field have also benefited people with the chronic subtype of CH who may have become refractory to the limited arsenal of pharmacological treatments available.

Over the years, the application of neurostimulation therapies has progressed from very invasive approaches, such as deep brain stimulation, to less invasive ones, namely occipital nerve stimulation [22] with promising outcomes in challenging-to-treat group of patients. Recently, the stimulation of the sphenopalatine ganglion (SPG), using a wireless microstimulator able to deliver on demand electrical therapy, has been shown for the first time to be effective in aborting and preventing CH attacks [23]. Despite these advances in the treatment of CH, there is an urgent need for policy makers to facilitate the implementation in clinical settings of new evidence-based treatments for chronic CH to help reduce the devastating burden of this condition [24–26].

In conclusion, the 2017 Cluster Headache Awareness day incorporated joint efforts, directed to the general public, governments, policy makers, healthcare professionals and the media, to emphasise the unmet needs of this underdiagnosed and mistreated condition. For far too long lack of awareness, misperception of its burden and lack of research funding have hindered developments in the understanding of the condition. Challenging these historical issues is our responsibility. Offering a brighter future for patients, our accountability.
